# Sydenham Society

**Published:** 1844-06-15

**Authors:** 


### SYDENHAM SOCIETY.

*Philadelphia*, 109 *S.* 10*th St.*,

June 6th 1844.

MY DEAR SIR,—May I request the favour of you to state, for the information of those who have become members of the Sydenham Society through my instrumentality, that I have this day received a letter, of which the following is a copy, from Dr. Jas. R. Bennett, the Honorary Secretary of the Society?

24 *Finsbury Place*,

May 1, 1844.

MY DEAR SIR,—I am afraid you will have thought us negligent of our American friends, but the press of matters requiring consideration previous to the anniversary meeting, and the arrangements necessary to be made with reference to the mode of transmitting subscriptions, have prevented my replying sooner to your communications. We have now arranged with Messrs. Baring, Brothers & Co., to have our American subscriptions transmitted to them through Messrs. Prime, Ward & King, of New York. You will therefore please to pay to Messrs. Prime, Ward & King, of New York, £1 1s. for every subscriber to the Sydenham Society. The Society will deliver the books to any address in London that you may direct, and will pay all charges for the remittance of money from Messrs. Prime & Co. But the carriage of the books will fall on the members. If, however, a number go together, we presume this will be very trifling, and will be easily apportioned to the different members. For the gentlemen whose names you have sent, I have reserved copies of the two works already issued, which will be despatched immediately on our receiving the subscriptions from Messrs. Barings.

The council are greatly obliged by your kind offer of services, and will be very happy to receive any suggestions in reference to works that you may think worthy of the attention of the society. Our second annual meeting has just taken place, and the society is prospering far beyond our expectations. Our Reports will soon be printed, when I will take care to send you a supply for distribution.

Believe me, dear sir,
Yours very respectfully
JAS. R. BENNETT,
Hon. Sec.

DR. ROBLEY DUNGLISON.

In accordance with the instructions contained in the above letter, I have this day transferred into the hands of Messrs. Prime, Ward & King the amount of subscriptions paid to me; and by the Caledonia steamer from Boston of the 16th inst., I shall inform Dr. Bennett thereof: instructing him as to the best mode of transmitting the publications of the Society to this city. These arrangements have necessarily given occasion to delay in the reception of the first publications of the society, which cannot exist in future.

I am, my dear sir, truly yours,
ROBLEY DUNGLISON.

Prof. R. M. HUSTON, Editor of the "Examiner."

